# A pediatric case report of Epiploic appendagitis presented with abdominal pain

**DOI:** 10.1016/j.ijscr.2020.03.033

**Published:** 2020-04-01

**Authors:** Ohoud Baajlan, Hotoun Bokhari, Khalid AlGhamdi, Mazen Zidan

**Affiliations:** a King Abdulaziz University, Jeddah, Saudi Arabia; b Department of Paediatric Surgery, King Fahad Armed Forces Hospital, Jeddah, Saudi Arabia

**Keywords:** Case report, Appendicitis, Epiploic appendagitis, Computed tomography

## Abstract

**Introduction:**

Epilopic appendagitis (EA) is an uncommon condition of abdominal pain caused by the local inflammation of the fat-filled peritoneal outpouchings due to torsion or thrombosis of its vessels leads to ischemia and gangrenous necrosis of the aappendages, as it can cause peritoneal irritation, acute ischemia, and fat necrosis.

**Case report:**

We present a case of epilopic appendagitis mimicking appendicitis of a 10 years old male, presented to the emergency department with severe right quadrant pain pointedly at the right lumbar area. Associated with constipation and nausea for once. Computed tomography (CT) scan with contrast was performed showing an ovoid fat structure with thin enhancing rim and surrounding inflammatory stranding as well as prominent lymph nodes at hepatic flexure, free fluid and no evidence of appendicitis. The patient was discharged with pain control medications.

**Discussion:**

Epiploic Appendages are mobile, pedunculated peritoneal out pouches. Considering its mobility and narrow pedicle appendages are disposed to torsion leading to appendagitis causing local inflammation, peritoneal irritation, acute ischemia, and fat necrosis. The patient’s main complaint would be a subacute lower abdominal pain, left-sided in 60–80% of cases.

**Conclusion:**

Early recognition of this condition is crucial to avoid an operation when unnecessary leading to prolonged hospital stays. The management is conservative with analgesic.

## Highlights

• Epiploic Appendages are mobile, pedunculated pouches.
• It’s a self-limited condition that could occur due to torsion or thrombosis of its vessels.
• The Patient’s complaint may be subacute lower abdominal pain, left-sided in in most cases.
• Early recognition of this condition is crucial to avoid an operation when unnecessary leading to prolonged hospital stays.

## 1 Introduction

Epilopic appendagitis (EA) is an uncommon condition of abdominal pain caused by the local inflammation of the fat-filled peritoneal outpouchings due to torsion or thrombosis of its vessels leads to ischemia and gangrenous necrosis of the aappendages, as it can cause peritoneal irritation, acute ischemia, and fat necrosis [1,2].

The patient’s typical presents with acute or subacute lower abdominal pain, left-sided in 60–80% of cases that is usually indistinct with appendicitis, diverticulitis or acute cholecystitis. White blood cell count and CRP are generally normal, though maybe mildly raised, infrequently it could be associated with fever, nausea, and vomiting [[3], [4], [5], [6]]. It’s a self-limited condition that is initially managed conservatively.

In this report, we present a case with abdominal pain who was diagnosed with EA. And was successfully treated with conservative management.

This report has been written in accordance with SCARE criteria guidelines for case reports [7].

## 2 Presenting case

In July 2019, A 10 years old Saudi male was brought to the emergency department by his parents complaining of abdominal pain.

The pain started 5 days before the presentation, it was generalized at first, then it became only localized to the right quadrant area, pointedly at the right lumbar area. The pain was severe with intermittent with an on and off in character, with no relieving factors and it increases with motion, not radiated to anywhere else, and this was his first episode of similar pain. It was associated with constipation and the patient also reported being nauseated once, with no vomiting, fever, night sweats, or loss of appetite.

Our patient has been diagnosed with asthma since he was 4 years old, controlled on Ventolin as needed, it has been a month since his last asthmatic attack. He has no previous admissions to any hospitals, and no previous surgical history as well. On clinical examination, the patient was in pain, (weight: 48.45 kg, Height: 141 cm and BMI: 25 kg/m^2^). All his vital signs were stable.

**Fig. 1:**
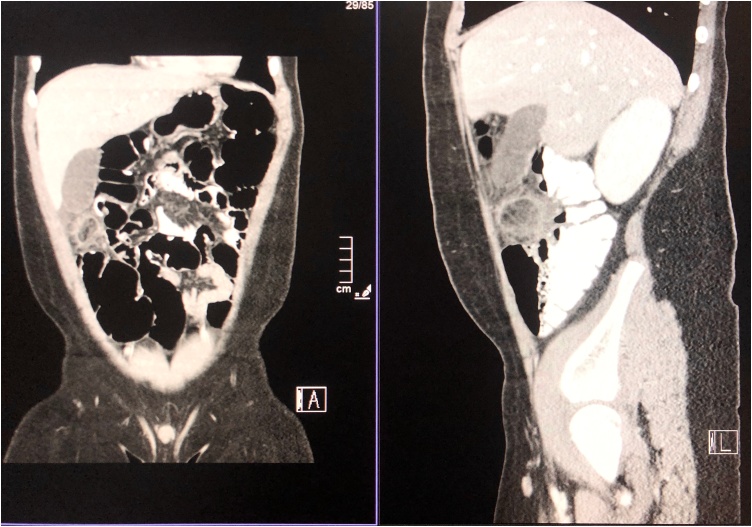
Abdominal CT scan with contrast showing inflammatory stranding and free fluid in the pelvis.

**Fig. 2:**
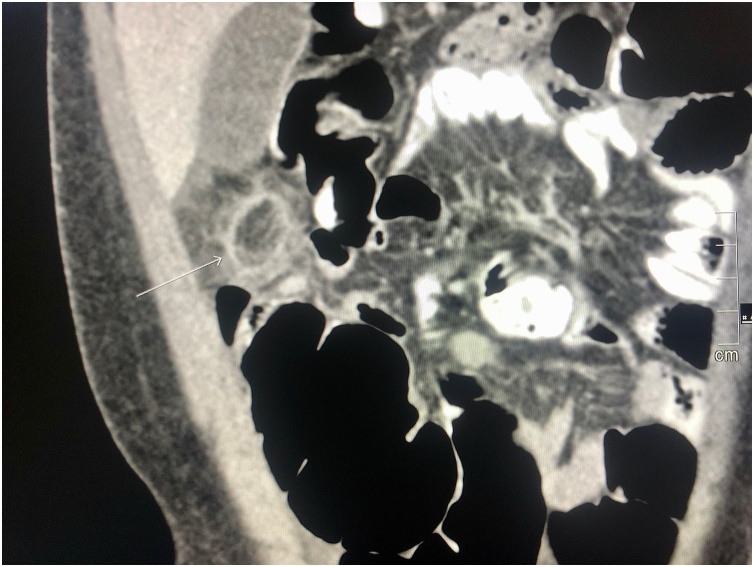
Abdominal CT scan with contrast showing an ovoid fat structure with thin enhancing rim and inflammatory stranding with free fluid in the pelvis.

**Fig. 3:**
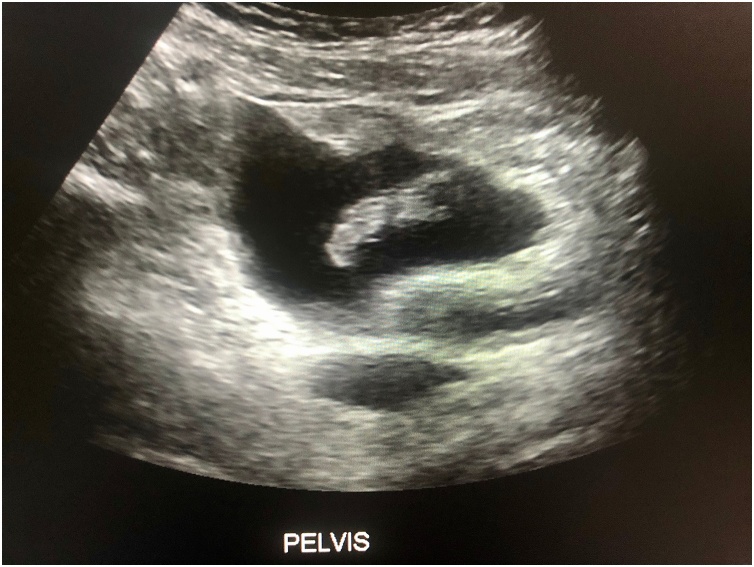
Pelvic ultrasound.

His abdomen was soft and lax, mild tenderness at the lumbar area, and the patient been asked to be NPO. In the absence of classical clinical symptoms of appendicitis, an Ultrasound and Abdominal Computed tomography (CT) scan with contrast was offered to rule out clinical suspicion of acute appendicitis or other pathology (Fig. 1, Fig. 2, Fig. 3). CT scan showed a normal appendix measured with a wall thickness of 4 mm. There was an ovoid fat structure measured 2.2 × 2.5 cm with thin enhancing rim and surrounding inflammatory stranding as well as prominent lymph nodes at hepatic flexure, free fluid was seen in the pelvis. The patient was discharged with pain control medications.

## 3 Discussion

Epiploic Appendages are mobile, pedunculated peritoneal out pouches developing from the serosal surface of the colon, the two most common sites for this to develop is the cecum and sigmoid. Regarding size, it’s 0.5–5 cm long with a thickness of 1–2 cm comprising of adipose tissue and peritoneal vessels. Its blood supply is from two arterioles from the vasa recta and a single venule [8]. Considering its mobility and narrow pedicle appendages are disposed to torsion leading to EA.

EA can arise at any age, pediatric cases are very rare with no gender preponderance unlike in adults it is most likely in the 4th and 5th decades with men gender preponderance [[9], [10], [11]]. It is more associated with obesity and high abdominal adipose tissue [3,[12], [13], [14]] although some studies reported no association [7,15].

The typical presentation is with acute or subacute lower abdominal pain, left-sided in 60–80% of cases. And on examination, there is localized tenderness with or without guarding and rebound tenderness. Due to the indistinct presentation of the complaints, patients are often misdiagnosed with other acute inflammatory diseases such as acute appendicitis, diverticulitis, or cholecystitis. Mostly patients are vitally stable with normal laboratory results but a mild leucocytosis and minor elevation of C-Reactive Protein in some cases as reported [8].

In the presenting case, a ten-year-old boy with a body mass index of 25 kg/m^2^ whose symptoms included abdominal pain, nausea, constipation and mild tenderness of the lumbar area on examination was found to have EA.

The diagnosis was achieved in the past through surgery, but currently, imaging studies showed high diagnostic yield. Normally epiploic appendages is not shown in imaging studies unless inflamed. CT is the diagnostic method of choice because of the distinctive appearance of EA as it appears as pericolonic oval-shaped mass with fat and soft tissue hyperattenuating rim corresponding to the inflammation and thick visceral peritoneal lining with periappendageal fat stranding. A hyperdense central dot or linear high density ‘central dot sign’ is some cases seen demonstrating a thrombosed vessel or hemorrhagic necrosis [[16], [17], [18]]. Similar clinical and CT findings can be seen with omental infarction, inflammatory disease like appendicitis and acute diverticulitis, and mesenteric panniculitis [8,9,16,17].

EA on ultrasonography manifests as a non-compressible hyperechogenic mass near the colon wall, with hypoechoic rim indicating the inflammation around the peritoneum with an absence of blood flow on Doppler US scan [8,16]. MRI can be helpful in the diagnosis of EA but it is rarely used [9,18,19].

EA is a self-limiting, benign disease that mainly managed conservatively with Nonsteroidal anti-inflammatory drug (NSAID) treatment. Nevertheless, EA can be followed by a variety of complications such as adhesions, obstruction, Intussusception and local abscess formation, as it also has the possibility to recur in some cases. Thus, failure of medical management is an indication for surgical intervention besides complicated EA like in obstruction and abscess formation [8,9,16,19].

In the current case, the patient was diagnosed by a CT with contrast and was managed effectively with conservative management and discharged from the hospital without complications.

## 4 Conclusion

These symptoms are not specific and could mimic appendicitis as in the presentation of this patient. CT with contrast was done to verify the diagnosis however the benign condition of appendagitis was confirmed via the finding of pericolonic ovoid mass with rim attenuation and pericolonic fat stranding. When venous thrombosis is the case a central hyperdense dot sign is also considered specific. Early recognition of this condition is crucial to avoid an unnecessary antibiotic, operation and prolonged hospital stays. The management is conservative with NSAIDs. Follow up CT is unnecessary, but if done could show a decline in the pouch size, calcifications could also be seen in old infarcts.

## Declaration of Competing Interest

The authors declare that they have no known competing financial interests or personal relationships that could have appeared to influence the work reported in this paper.

## Sources of funding

There are no sources of funding.

## Ethical approval

Approval has been given by King Fahad Armed Forces Hospital Research and Ethics Committee, Jeddah, Saudi Arabia has approved this study. (Ethical approval Number: REC 326).

## Consent

Written informed consent was obtained from the patient's parents for publication of this case report and accompanying images. A copy of the written consent is available for review by the Editor-in-Chief of this journal on request. This report does not contain any personal information that could lead to the identification of the patient.

## Author contribution

Ohoud Baajlan: First and corresponding author, writing – Original Draft, Review & Editing and data collection.

Hotoun Bokhari: Contributed Study concept, data collection, interpretation, writing – Original Draft.

Kahlid AlGhamdi: Contributed to the study concept, and Writing – Original Draft and methodology.

Mazin Zidane: Supervised the writing of the manuscript and approved the final manuscript.

## Registration of research studies

1. Name of the registry: King Fahd Armed Forces Hospital – Jeddah.
2. Research and Ethics Committee.
3. Unique identifying number or registration ID: REC 326
4. Hyperlink to your specific registration (must be publicly accessible and will be checked):

## Guarantor

Dr. Mazen Zidan, MD

Pediatric Surgery,

King Fahd Armed Forces Hospital,

Saudi Arabia, Jeddah.

## Provenance and peer review

Not commissioned, externally peer-reviewed.
